# Notes of the Congress

**Published:** 1893-10

**Authors:** 


### NOTES OF THE CONGRESS.

Mr. Ernest Hart, the learned and distinguished editor of the *British Medical Journal*, delivered an address at the Pan-American Medical Congress, at Washington, the subject of which was The Ethics of the Medical Profession. In the course of this remarkable address, Mr. Hart animadverted upon consultations with homeopaths, and, if he is correctly reported in the daily newspapers, characterized these practitioners as quacks, supporting his assertion by a reference to Dr. Johnson's definition of "quack." It is true that the published abstract does not read quite that way, but even the revise makes Mr. Hart come dangerously near the line.

It seems a pity that the otherwise harmonious proceedings of the Congress should have been disturbed by such an address. However much we may be willing to tolerate a discussion on ethics in our local societies, we have always held that a national or international congress was not the place to deal with this question. It applies entirely and totally to the local societies, and Mr. Hart makes a mistake when he comes to America and drags in such questions gratuitously, as he did both in Milwaukee and in Washington. We hope our homeopathic friends will not be disturbed by Mr. Hart's dogmatic assertions, or conceive any dislike to the Congress on that account, for we beg to assure them that it was entirely foreign to the purposes of the Congress to have any such matter interjected into its proceedings. We hope it will be excluded from the Transactions.

---

The foreign delegates, after the adjournment of the Congress, made a special tour, as the guests of the United States, under charge of Dr. S. S. Adams, of Washington, chairman of the committee of arrangements. This tour included Philadelphia, New York, Boston, Niagara Falls, Detroit, Cincinnati, St. Louis, and Chicago. They were specially entertained in these several cities by the medical profession, and their visit to the several points of interest will ever be a memorable one. At Chicago, after visiting the World's Fair, the members separated for their several homes, apparently much delighted with the visit they had made to this country.

---

While the Pan-American foreign delegates were in Philadelphia, and during their visit to the grounds and buildings of the University, Dr. Charles A. L. Reed, Secretary-General, was pleasantly surprised by the presentation of a silver salver, on which was inscribed the following: "Presented to Dr. Charles A. L. Reed, of Cincinnati, Ohio, Secretary-General, by the members of the First Pan-American Medical Congress, Washington, D. C., September 4–8, 1893, to commemorate the brilliant success—largely due to his faithful and devoted efforts in its organization—of that important occasion, when for the first time representatives of the medical profession of the Western Hemisphere met in council for the advancement of science and the promotion of the public health."

Dr. Pepper, President of the Congress, made a happy speech as he uncovered the salver, and Dr. Reed, in accepting it, replied in a most felicitous manner. Dr. A. M. Owen, Treasurer of the congress, then took Dr. Pepper by surprise, by presenting him with an ivory gavel, appropriately inscribed, commemorative of the congress. Dr. Owen's speech was in his usual happy vein, and served to loosen the tongues of several delegates, who made speeches in a multiplicity of languages.

---

"Ernest Hart, F. R. C. S., D. C. L., Editor of the *British Medical Journal*, Dean of St. Mary's Hospital." This is the way, says Dr. Hammond in the *New York Medical Journal* of September 16th, that it appeared on the register at the Arlington hotel. Then Mr. Hart proceeded to read a lecture to the American medical profession on ethical morals, in which he deprecated so-called advertising in the newspapers, through the publication of interviews and photographs, and especially did he consider it wicked to consult with homeopaths. A very Daniel come to judgment!

---

"The American Medical Editors' Association" held a banquet at the Arlington hotel on Monday evening, September 4, 1893, which was the inaugural feast at the congress. The President, Dr. C.

H. Hughes, of St. Louis, occupied the post of honor and made a brief but spirited address of welcome. Then he turned the meeting over to Dr. I. N. Love, the talented editor of the *Medical Mirror*, who acted as toast-master. Among the regular toasts were: "The President of the United States," responded to by Hon. J. Sterling Morton, Secretary of Agriculture; "The Secular Press," responded to by Hon. Frank Hatton, of the *Washington Post;* "The American Medical Association," by the President, Dr. J. F. Hibberd, of Richmond, Ind.; "The Pan-American Medical Congress," by the President, Dr. William Pepper; "The Medical Press," by Dr. Hobart A. Hare, editor of the *Therapeutic Gazette;* "The Surgeon-General of the Army," in the absence of Surgeon-General Sternberg, was responded to by Surgeon-General William A. Hammond, retired; "The Surgeon-General of the Navy," by the Hon. John B. Henderson, of Missouri, President of the Pan-American Congress of four years ago; "The Journal of the American Medical Association," and "The Public Health," by Dr. John B. Hamilton, Surgeon of the U. S. Marine Hospital Service and editor of the *Journal.*

Among those who made voluntary speeches were Dr. Phillipot, of Jamaica; Dr. A. M. Owen, of Evansville, Ind., and Dr. Alonzo Garcelon, ex-Governor of Maine. Major Stofer, the well-known Washington correspondent, and Mr. Seabrooke, the actor, entertained the company between speeches with musical selections and recitations. This proved one of the most entertaining of the social features of the Congress.

---

THE efforts of the Medical Society of the State of New York, in advancing the cause of higher education, is meeting with some appreciation, as will be noted by the following, which we clip from the Chicago *Medical Standard:*

Whatever be the opinion of the New York State Medical Society anent its position on the code, it is clearly doing more to elevate the status of the profession and the individual physician than any other State society. Through its efforts, the medical institutions of the State have been lifted from the slough of "baseness" and "politics." No one can be selected as medical officers except from such as have passed an examination. A tenure-of-office act secures them in their position. The excellent medical practice act of that State was originated by the State Medical Society. It, and all other reforms, have been bitterly fought by a heterogeneous crowd of allies, among whom patent medicine "fakirs" and old codeite professors loom up prominently.

